# Current use of wild plants with edible underground storage organs in a rural population of Patagonia: between tradition and change

**DOI:** 10.1186/s13002-015-0053-z

**Published:** 2015-09-25

**Authors:** Juan José Ochoa, Ana Haydee Ladio

**Affiliations:** Instituto de investigaciones en Diversidad Cultural y Procesos de Cambio, San Carlos de Bariloche, Río Negro, Argentina; Instituto de Investigaciones de Biodiversidad y Medio Ambiente, San Carlos de Bariloche, Río Negro, Argentina

**Keywords:** Ethnoecology, Age, Gender, Family structure, Ethnic self-determination, Marginal food, Functional food

## Abstract

**Background:**

Edible plants with underground storage organs (USOs) are neglected resources. We studied the local ecological knowledge edible plants with (USOs) in rural populations of North-Patagonia in order to establish how people are utilizing these plants. Some aspect of corpus-praxis-cosmos complex associated to the local ecological knowledge was documented and discussed. In addition, variation in this ecological knowledge due to age, gender, family structure, ethnic self-determination was also evaluated.

**Methods:**

Semi-structured interviews were conducted with 51 inhabitants in order to study the relationship between the current use of plants with USOs and the age, sex, family group composition and ethnic self-identification of interviewees. In addition, the Cultural Importance Index for each species was calculated.

**Results:**

The current richness of known species in these populations is a total of 9 plants. Plants with USOs tend to be used more frequently as the age of the interviewee increases. Women and men showed no differences in the average richness of species cited. The interviewees who share their homes with other generations use these plants more frequently than those who live alone. Our results indicate that the interviewees who identified themselves as belonging to the Mapuche people use these plants more frequently.

**Conclusion:**

For the Mapuche people, wild plants have constituted material and symbolic resources of great importance in their historical subsistence. In addition, they are currently being redefined as elements which present a connection with ancestral practices, produce a strong relationship with the ‘land’, and become markers which identify the ‘natural’ (historical) ways of their people; these are key elements in the current political processes of identity revaluation. This research is valuable to stimulate cultural revival and health promotion programs in the communities with their own local, cultural food.

## Background

The use of edible wild plants forms part of the cultural traditions of rural and suburban societies throughout the world [1]. Plants with underground storage organs (USOs) are one kind of this edible resource and involves a set of species adapted to environmental factors (drought, lack of light, grazing and fires, amongst others) [2]. This category includes bulbs, tubers, rhizome, corms and/or true root thickenings, all structures which tend to act as water, carbohydrate and protein reservoirs [3]. The use of USOs was documented since prehistoric times in different part of the world. For example, throughout the Holocene period different archaeological sites in the Great Plain of North America shows the presence of edible geophytes associated with cook stone technology [4]. In the archeological site of Monte Verde (Chile) one of the first human occupation en South America dating from the end of Pleistocene, are common the presence of edible tuber of *Solanum maglia* and digging stick [5]. The use of edible roots is also documented in the site Calowanie date from the paleolithic and mesolithic times of the Polish plain [6].

The use of these edible wild plants were also present in historic and contemporaneous rural and indigenous societies in all the world [7–12]. In some societies these plants represent the principal source of carbohydrate. For example, in some part of western North America, indigenous groups use tubers of *Sagittaria* spp, *Camasia* spp and at least 25 species of plants with USOs in the preparation of favoured staple food [13]. The use of tubers of *Dioscorea* species in Africa [8, 14], is another example of the place of USOs in the food culture of different part of the world. In other cases the use of USOs are not staple but complement other foods or have the function of emergency resource in time of food crisis (wars, famine periods) [15].

In Patagonia the gathering and use of wild plants is a practice that has contributed to the subsistence of pre-Hispanic [16] and post-Hispanic [17, 18] populations in different geographical areas and periods of time. Since at least the 16th century the practices of gathering wild plants with underground storage organs for nutritional reasons have a good register [19, 20]. An analysis carried out of written sources dating back to the 16th century and continuing up to the present time [20] shows that there are around 50 species of plants with edible USOs, whose geographical use distribution is concentrated around arid regions of the Patagonian steppe. During the period of post-colonial contact the use practices of this kind of species seem to have followed at least two contrasting models of use [20]. The first model, which we will call “*intensive use* of USOs”, suggests that for the 16th and 17th centuries some of these plants, along with the meat of the *guanaco* (*Lama guanicoe*) and *choique* (*Rhea pennata*), constituted the main diet of the known indigenous populations [21, 22]. The second model, in contrast, the “*marginal use* of USOs” describes a very different situation, in which these species occupy a marginal place in the diet and were used in social or ecological contexts of scarcity of other food sources [20]. This strategy is evident in the later periods of European colonisation (18th to 20th centuries) and is probably related to sociocultural transformations, such as the forced displacement of indigenous peoples to other regions, impeding access to environments they had traditionally used, or the intervention of the State in the local diet through the incorporation of processed foods [23]. According to some authors [24, 25], changes in ecological dynamics, such as the incorporation of new exotic resources, may also have favoured changes in the diet of native populations.

As with other cultural practices associated directly with the environment, the gathering and use of wild plants occurs in complex contexts where social and ecological factors are closely linked [26]. In this sense the study of the interaction between people and wild plant with USOs needs a scientific approach that takes into account this multidimensional complexity. Ethnoecology is a scientific perspective which in recent years has explored human practices related to the use of wild resources, contextualising them in socioecological systems [27]. This discipline proposes that the practices of gathering and using plants (praxis) occur and make sense when we consider them in association with a body of knowledge (corpus) and beliefs (cosmos). This association may be described as a network within which each of these dimensions provides feedback to all the others. On the one hand, the processes of transmission and learning of plant knowledge gives shape, over time, to a shared, differentiated set of knowledge (corpus) and beliefs (cosmos) on the subject. Use is itself an experience that reaffirms the learned properties, and thus influences individual knowledge, which is then projected on the group corpus through socialisation. These three dimensions are reflected in what some authors have called local ecological knowledge [26], which refers to this set of practices; know how, values and beliefs which human individuals and groups develop with respect to their environment.

Several studies have shown that local ecological knowledge on wild plants is dynamic and can vary due to multiple social, temporal and environmental factors [28]. For example, it has been documented that knowledge of wild plants is positively correlated with age [29], since learning processes take place not only in a time sequence going from childhood to adulthood, but also as time goes on, when experiences of use may be more frequent [30]. In rural contexts, social gender roles relating to domestic chores and activities associated with agriculture and livestock may have an influence on interaction with the wild elements in the environment [31]. For example, it has been documented that in certain cases, women who are in charge of family health know more about medicinal wild plants, whereas men who are involved in work related to construction know more about plants used for this purpose [31].

It has been proposed that the permanence of these practices may have a positive correlation with the family structure of the person who uses the plants, since environments where there is inter-generational interaction favour the need for these plants and promote opportunities for learning about them. For example, Ochoa et al. [32] found that in a rural population of northwest Patagonia, people who lived alone used lower richness of wild species compared to those who lived with children and/or grandchildren.

The cosmovision (cosmos), that is, the way of perceiving, conceptualising and valuing the world around us [33], is another aspect that is fundamental to the understanding of variations which may occur in the use of plants, particularly in societies where people of different backgrounds live together. Such is the case in many contemporary rural societies, where the cosmovisions of native peoples, religious metaphysics and modern scientific visions coexist, and influence interaction with the environment in different ways. At the present time, the rural areas of Patagonia are populated (either widely dispersed or concentrated in small settlements) by people who identify themselves as belonging to native peoples (Mapuche and/or Tehuelche), who, whether organised into communities or not, depend on extensive livestock farming as their main living. The Mapuche cosmovision, as with the other American indigenous populations, is characterised by its harmonic relationship with the elements which make up the world (human, non-human, spirits, etc.) [34]. The land possesses a sacred aspect, and human acts may alter the equilibrium and the health of the surroundings if the harmonic relationship between the parts is affected. Plants have held an important place in the cosmovision of these peoples, as part of their diet, medicine [35] and various religious aspects [34], and they continue to form part of current cultural practices [19, 30].

In order to explore local ecological knowledge of plants with edible USOs in rural populations of northwest Argentine Patagonia, in this work we propose to: 1) document the richness of known plants with USOs and the different ways they are used (corpus) in four rural communities in Patagonia, 2) identify the ways they are taken advantage of (praxis) and evaluate whether these are part of a “principal” or “marginal” use strategy, 3) identify aspects related to the current cosmovision (cosmos) of these plants, 4) analyse the cultural importance of each species at a regional level, and 5) explore the relationship between current use and sociocultural variables such as age, gender, family group structure and ethnic self identification.

Our principal hypotheses refer to the fact that plants with USOs, studied from the corpus-cosmos-praxis complex, constitute distinctive resources in the Patagonian communities, and reflect current traditions, but also processes of change. This work reveals the level of articulation of multiple factors necessary so that knowledge constructed in a multidimensional fashion can continue to be recreated in new social and economic contexts. In addition, this research is valuable to establish salient indicators of changing practices and to stimulate cultural revival, environmental conservation and health promotion programs in the communities with their own local, cultural food.

## Methods

### Study area

**Fig. 1:**
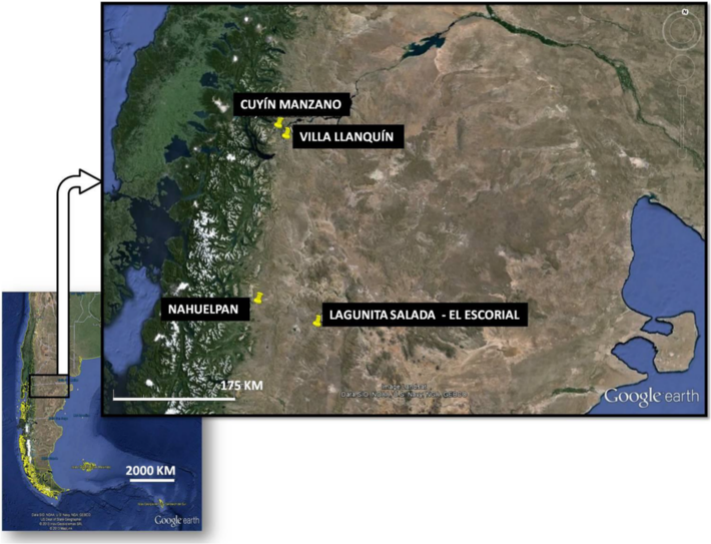
Study Sites: Cuyín Manzano (Prov. Neuquén); Villa Llanquín (Prov. Río Negro); Nahuelpan (Prov. Chubut). Image created using Google Earth © Google Inc. (visited 12/2013)

The work was carried out in 4 populations of north western Argentine Patagonia, which lies on an environmental gradient that goes from a severely arid steppe region (Lagunita Salada – El Escorial) to a steppe region closer to the Cordillera, with remnants of conifer forests (*Austrocedrus chilensis*) (Villa Llanquín), then to another region that is also ecotonal between steppe and forests of *Austrocedrus chilensis* and *Nothofagus* spp. (Nahuelpan and Cuyín Manzano) (Fig. 1, Table 1). Despite their historical distinctions, they have things in common with all the rural populations in the region, such as the historic predominance of livestock breeding, ancestors from native populations and current diversification into other economic activities such as tourism [36]. It should be clarified that Villa Llanquín is organised in such a way that a population nucleus can be distinguished which is in the process of urbanisation (blocks, public services, immediate access to the market), and a population with more rural characteristics (homes several km distant from each other, with no public services) which is spread out over 40 km to the north, south and east of this nucleus. In this study we worked with inhabitants who lived within the latter context.

**Table 1:** Climatic, phytogeographical and social features of the study populations [37, 38]

	Rural villages		
	Cuyín Manzano	Villa Llanquín	Nahuel Pan
Annual average temperature (°C)	7.4	8	8
Annual average rainfall (mma)	600	500	400
Phytogeographical environmental	Ecotone between Andean Patagonian Forest and Subantarctic Steppe	Steppe - Andean Patagonian Forest fragments	Patagonian Steppe, Andean Patagonian Forest (in the west)
Dominant plant communities	Forest (*Nothofagus pumilio*, *Austrocedrus chilensis*, *Maytenus boaria*, *Lomatia hirsuta*) herbaceous and shrub steppe (*Stipa* spp; *Mulinum spinosum* and *Senecio* spp.)	Herbaceous and shrub steppe (*Stipa* spp; *Mulinum spinosum* and *Senecio* spp., Nassauvia spp.) and Forest fragments of *Austrocedrus chilensis*, *Maytenus boaria*, *Lomatia hirsuta*.	Herbaceous and shrub steppe (*Poa, Stipa* and *Festuca*; *Mulinum spinosum, Nassauvia spp., Berberis heterophylla, Adesmia campestris, Nardophyllum obtusifolium, Azorella monantha, Senecio filaginoides, Corynabutilum bicolor* and *Schinus roigii*), Forest of *Nothofagus pumilio* and *N. Antartica*
Population	50 people	113 people	60 people
Institutions	Primary school, Health care service, Police, Local Council, Park Ranger	Primary school, Health care service, Police, Local Council	Primary school, Train station
Economic activities	Tourism, Big game hunting, public and private employment, extensive livestock breeding.	Extensive livestock breeding horticultural production, tourism, public employment.	Extensive livestock breeding, tourism.

### Data collection

Information on the context and characteristics of the project was given to the social leaders of each community, and the informed consent of each informant was obtained [39]. In addition, informants consented to document and publish the results of the study. This informed consent was verbally obtained prior to conducting interviews according to the ethical guidelines suggested by the Code of Ethic of the International Society of Ethnobiology. The homes visited were randomly selected. Open talks, semi structured interviews (*N* = 51) and participant observation, involving tours of the gathering sites of plants with edible USOs, were carried out. One family member was chosen in each home where the inhabitants agreed to participate in the study. The whole family was asked to consider who would be the interviewee, based on their plant knowledge. In Cuyín Manzano 16 people were interviewed (representing 90 % of the homes in the village), 10 men and 6 women, average age: 54 ± 15; in Villa Llanquín, 18 people (70 % of homes in the non-urbanised area), 11 men and 7 women, X: 61 ± 11; in Nahuelpan, 7 people (33 % of homes), 4 men and 3 women, X: 63 ± 16; and in Lagunita-Escorial 10 people (20 % of homes), 5 men and 5 women, X: 54 ± 10. Of all interviewees, 24 shared their homes with more than one generation, whereas 27 lived alone (8) or with their partners (19).

While 26 interviewees identified themselves as Mapuches, 25 considered themselves Creoles. During the interviews socioeconomic characteristics were surveyed (age, gender, number of inhabitants in the home and ethnic self identification). Each informant was asked about their knowledge of plants with edible USOs. For each of the species mentioned, questions were asked as to frequency of use, gathering methods and consumption. In addition, during the open interviews informants’ perceptions and evaluation (cosmovision) of the plants’ quality in terms of nutrition, functionality and/or relation with the environment were recorded, as well as memories of use in the past by ancestors. Tours of the gathering sites mentioned were carried out with informants. Field notebooks and audio recordings of the interviews are available in the *Instituto de Investigaciones en Diversidad Cultural y Procesos de Cambio* (CONICET-UNRN) laboratory. Samples of plants with USOs were herborised under the label number indicated in Table 3 and placed in the ECOTONO laboratory of the *Instituto de Investigaciones en Biodiversidad y Medioambiente* (INIBIOMA).

### Data analysis

The data obtained from this study were analysed in two ways: i) qualitatively, through discursive analysis of the interviews and participant observation records, ii) quantitatively, by re-categorisation of the information, estimation of frequencies and the use of the indices detailed below, which were analysed statistically [39]. To compare the species similarity between this work and published in a similar study carried out in the region [19] we used the Jaccard Similarity Index (JSI). This index take account the presence/absence of plants in a sets of data and expresses the number of species in common with respect to the total number of species. JSI = (c/a + b + c) × 100, where “c” is the number of common species between the two set of data, “a” is the number of species that only found in the set of [19]`s data, and “b” is the number of species that only found in the set of our data (the present research). Table 2 shows the categorization carried out for the variables which were quantified and used in the calculation of the cultural importance index. Calculation of the cultural importance (**CI**) of each species at regional level was based on the index proposed by Gonzáles et al. [40] and modified according to the following criteria: On the one hand, the variable “number of settlements where it is known” (**S**) was added, given that we are interested in knowing the cultural importance at regional level, rather than local importance as used in the work of Gonzáles et al. [40]. The original variable “part used as food” was not included since for plants with USOs the underground storage organ is always the edible part used.

**Table 2:** Categorization of variables quantified and used for the construction of indices and statistical correlations

Sociocultural variables	Category
Gender	Women (0), Men (1)
Family group living in the house	One generation - alone, with partner or brother/sister - (1), more than one generation, children, grandchildren, grandparents - (2)
Ethnic self recognition	Non- indigenous/Creoles (0); Indigenous/Mapuche (1)
Variables related to use practice	
Richness of species used	Never used (0), one per year (1), n species per year (n)
Variables related to Cultural Importance Index (IC)	
Total number of settlements where species cited (S)	One (1), Two (2), Three (3), Four (4)
Relative frequency of cites (number of people that know the plant/total number of interviewees (*N* = 51) (RFC)	0.019 (minimum value if just one person mentions the plant); 1 (maximum value if all the people mention the plant)
Frequency of underground storage organs (USOs) harvested per year (UF)	No USOs harvested per year (1); n USOs harvested per year (*n* + 1)
Multiple use recognized (MU)	Single use (1), n uses (n)
Current use (CU)	Never used (1), Used in childhood (2), Used in the last year (3)
Management (M)	Harvested (1), Cultivated (2), Harvested and cultivated (3)
Flavour (S)	No mention (1); bitter-strong (2), sweet (3)
Forms of consumption (T)	Raw in situ (1), Raw ex situ (2); Some kind of preparation and ex situ consumption (3)
Commerce (C)	Without commerce (1), Under commerce (2)

The category “medicinal use” in the original index was replaced by “multiplicity of use”, since various species mentioned by our informants were assigned other uses apart from food and medicine. The “exclusivity of acquisition” and the “method of acquisition” variables were also removed since for this group of species our informants mentioned exclusively occasional use and acquisition by gathering. In addition, in order to increase the scale of this index we divided the product of these variables by 100 instead of 10000. The resulting cultural importance index for each species takes into account the number of settlements where it is known (**S**), the relative cite frequency (**RCF**), frequency with which the species is used (**UF**), multiplicity of use (**MU**), current relevance of use (**CU**), type of management (**M**), perceived flavour (**F**), type of consumption (**T**) and commercialisation possibilities (**C**) (Table 2). The UF value represents the underground storage organs (USOs) harvested per year. The variables **RCF**, **UF**, **MU**, **CU**, **M**, **F**, **T** and **C** express the average number of cites for each species.

$$ \mathbf{C}\mathbf{I} = \left(\mathbf{S}\times \mathbf{R}\mathbf{C}\mathbf{F}\times \mathbf{U}\mathbf{F}\times \mathbf{M}\mathbf{U}\times \mathbf{C}\mathbf{U}\times \mathbf{M}\times \mathbf{F}\times \mathbf{T}\times \mathbf{C}\right)/100 $$

With respect to analysis of the relationship between the use frequency of plants with USOs and sociocultural variables, the frequency which these species were used per person was correlated with the age of each of the informants (Spearman correlation, *p* < 0.05); variation due to gender, informant’s family group structure and ethnic self identification was analysed using the Mann Whitney Test (*p* < 0.05). Bonferroni correction was used to counteract the problem of the multiple and simultaneous comparisons by testing each individual hypothesis with the formula: *p* values ≤ α / k, with k = number of individual hypothesis were tested in this study). For example, our investigation was testing four hypotheses (about age, gender, ethic self-recognition, and family group) with a desired α = 0.05, then the Bonferroni correction tested each individual hypothesis at α = 0.05/4 = 0.0125.

## Results and discussion

### Richness of known plants with USOs

**Fig. 2:**
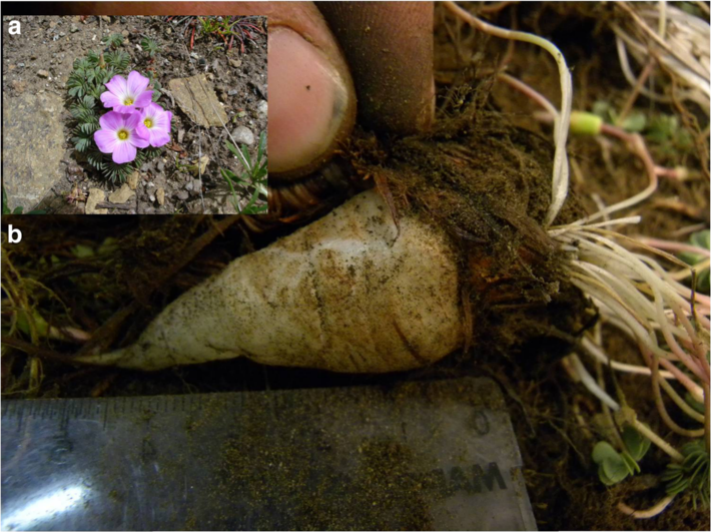
**a** General aspect of *Oxalis adenophylla*. **b** Edible root of *Oxalis adenophylla*

**Fig. 3:**
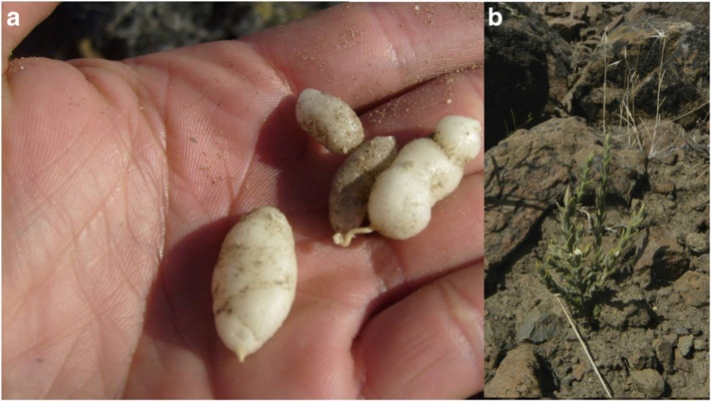
**a** Edible tuber of *Arjona tuberosa*. **b** General aspect of *Arjona tuberosa*

The accumulated richness or *corpus* relating to plants with USOs in the 4 communities studied was 9 species (Table 3), plus 2 ethnospecies which could not be scientifically determined. Of these, 7 species are native to Patagonia and 2 are exotic, corresponding to 7 botanical families with Apiaceae showing the highest richness. Six species were represented exclusively because of their edible underground storage organs, while for another 3 species up to 3 use categories were mentioned (Table 3). The species with the highest relative cite frequency (RCF) were *Oxalis adenophylla* (Fig. 2a) and *Arjona tuberosa* (Fig. 3a) (Table 3). On average each informant knows 2 species (max.: 5, min.: 1). This richness is similar to that found in other arid zones of the region and the world. For example, in Anatolia (Turkey) the people use 4 wild bulbs of the family Iridaceae [41]. For Mongolian shepherds a richness of 12 bulbs was documented, principally belonging to the family Liliaceae [42], while the Pumé of Venezuela use 18 different species [43].

**Table 3:** Richness of plants with edible underground storage organs known in the present study; P (Settlement where the species were cited): Cuyín Manzano (CM, *N* = 16), Villa Llanquín (VLL, *N* = 18), Nahuel Pan (NP, *N* = 7), Lagunita Salada-El Escorial (LE, *N* = 10); RFC (Relative frequency of mentions); MU (Multiplicity of uses): Medicinal (M), Edible (E), Ornamental (O), Fodder (F), Other uses (Ot); IC (Cultural Importance Index); OB (biogeographic origin): Native (N), Exotic (E)

Species/Botanical family/popular names cited/Label N°	P	RFC	MU	IC	OB
*Oxalis adenophylla*/Oxalidaceae/cuye/JJO 001, JJO 002, JJO 003, JJO 004	CM-VLL-NP	0.745	M, E, O	2.295	N
*Arjona tuberosa*/Santalaceae/shaquil, papita del piche/JJO 005, JJO 006, JJO 007, JJO 008	CM-VLL-NP-LE	0.568	E	0.951	N
*Diposis patagonica*/Apiaceae/yocon/JJO 009, JJO 010	VLL-NP	0.235	E	0.690	N
*Pastinaca sativa*/Apiaceae/pana, nabo silvestre/JJO 011, JJO 012	CM-VLL	0.058	E	0.185	E
*Tropaeolum porifolium*/Tropaeolaceae/batata, chagual/JJO 013	LE	0.098	E	0.141	N
*Helianthus tuberosus*/Asteraceae/papa del chancho/JJO 014	VLL	0.058	E, F	0.139	E
*Tristagma patagonicum*/Alliaceae/estrellita, cebollita/JJO 015, JJO016	CM-VLL-NP	0.117	E	0.123	N
*Azorella monantha*/Apiaceae/leña de piedra/JJO 017	LE	0.058	E, L, Ot	0.055	N
*Juncus* sp./Juncaceae/junco/JJO 018	NP	0.058	E	0.019	N

In a similar study carried out by Ladio [19] in three Mapuche communities in the north of Neuquén province in Argentina, situated some 500-1000 km from the populations in this work, it was found that the inhabitants (*N* = 98) know a richness of 12 plants with edible USOs. The richness similarity between this work and the present study was low (JSI = 19,23 %). This indicates, on the one hand, the importance of these species on a regional scale, but on the other hand, the particularities and lack of continuity observed in the way each population knows these plants. For example, *Oxalis adenophylla* is not mentioned for its edible root (Fig. 2b) in the Ladio work [19], but is known in those areas for its medicinal properties [44]. In contrast, this species appears as one of the principal plants with USOs in our study. Moreover, all the plants mentioned by Ladio [19] are native species, while at least 2 exotic species were mentioned in the communities we worked with, which also reflects the particular way of interacting with and knowing about the flora in each area, perhaps in relation to the different cultural backgrounds of these communities. In line with this, in the Mapuche community study by Ladio [19], the higher richness of plants with USOs, exclusively native species which in many cases maintain their Mapudungun phytonymy, could be indicative of higher maintenance of certain distinctive characteristics of the Mapuche culture in these populations compared to the ones involved in the present work.

### Plants with USOs: use practices

**Fig. 4:**
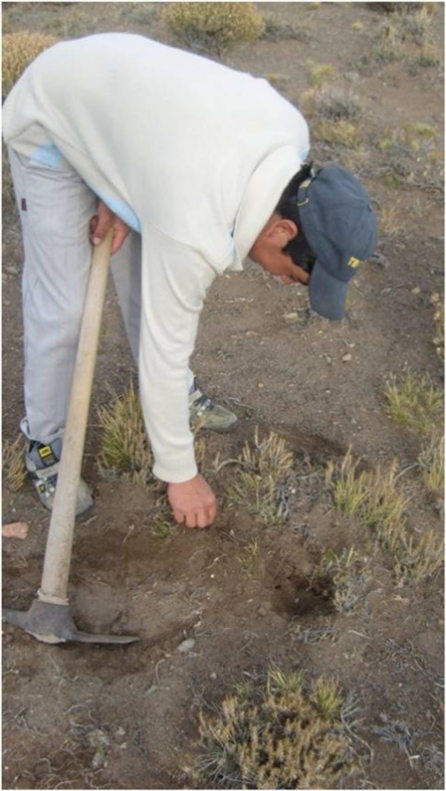
Inhabitant of Lagunita Salada digging up *Arjona tuberosa* tubers

**Fig. 5:**
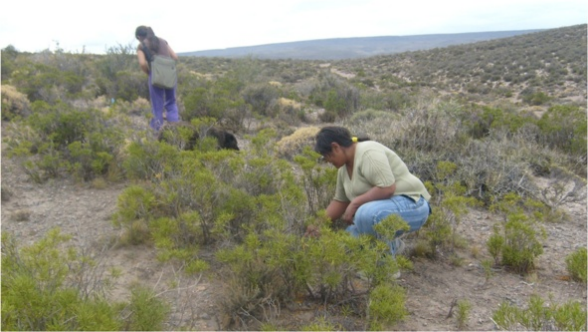
Inhabitant of El Escorial digging up *Tropaeolum porifolium* tubers

Of the inhabitants interviewed, 50 % consume plants with USOs at least once a year (Figs. 4, 5). The species currently used because of their storage organs, although with different frequencies, are *Oxalis adenophylla, Arjona tuberosa*, *Diposis patagonica*, *Tropaeolum porifolium* and *Azorella monantha*. The remaining 45 % of cites referred to consumption of the plants many years before, mainly in infancy (all the species were cited as having been used in the past). Only 5 % of the total cites of plants with USOs corresponded to people who knew the species (*Oxalis adenophylla* and *Arjona tuberosa*) but had never consumed it. Of all cites (*N* = 107), 64.5 % referred to the use of plants with USOs in infancy, 29.9 % to current use and 5.6 % to plants which had never been used. Gathering frequency at the present time (*N* = 32) is an average of 1.32 underground organs per year (SD: 0.75; max: 4, min: 1).

The principal method of consumption of plants with USOs is raw, in situ (100 % of cites). To a lesser extent, other ex situ methods were also mentioned, restricted to *Diposis patagonica*, *Tropaeolum porifolium* and *Arjona tuberosa*. (18 % of cites). These cites refer to memories of past practices carried out by parents or grandparents, in which the organs gathered were taken home and eaten either raw, seasoned (*Diposis patagonica*), cooked together with vegetables (*Diposis patagonica*, *Tropaeolum porifolium*) or left for some hours to soak in milk before being eaten along with the milk (*Diposis patagonica*, *Arjona tuberosa*).

Two types of management practice are found to be associated with the set of plants with USOs; gathering of wild specimens and cultivation. Gathering in the wild state is carried out for all species with the exception of *Helianthus*, which is always cultivated. The informants who cited the use of *Pastinaca* mentioned that in the past the plant had been exclusively cultivated, but that at the present time, in contrast, it is not cultivated, but grows wild. *Oxalis adenophylla* was the only native species which, in addition to being gathered in wild populations, is also cultivated. This practice is little known, carried out by only two inhabitants of Villa Llanquín. It consists in transplanting bulbs from wild populations to sites close to the houses. These plants are cared for in a similar way to plants in a vegetable garden; that is, they are planted in places where animals have no access and tend to be watered along with other garden plants. This is a new practice since both interviewees have been carrying it out for only the last four years, and they both relate this practice to the beauty of its flowers and the need to have easy access to this plant, which in its wild state tends to be found in distant sites. Experimentation with its cultivation, and memories of their parents doing it was another reason given for carrying out this practice. Finally, it should be noted that in this settlement one interviewee mentioned that every year they gather leaves of this species and prepare a medicinal *tortilla* which they sell at a local fair, *la fiesta del puestero* (The Ranch Hand Festival), which takes place every year in February in Junín de los Andes (Neuquén province).

As mentioned previously, three species are recognised for their multiplicity of use (Table 3). *Oxalis adenophylla* is known and used principally for the medicinal properties of its leaves, which are used to treat fever, and for its edible roots that are consumed like snack [44]. To a lesser extent, this plant is appreciated locally for its ornamental characteristics. *Azorella monantha,* whose edible root is currently used by only one person, is appreciated and used for firewood. Another current use registered is to employ the ashes produced by the plant when it is burnt for the cooking of bread. This traditional cooking method called “rescoldo” consists in wrapping the dough in ash, which favours the cooking process and gives it a particular flavour. Finally, the tubers of *Helianthus tuberosus,* while not currently consumed by settlers, are used as a food source for domestic pigs.

From the inhabitants’ point of view, the gathering and use of these species is a secondary activity, practiced at the same time as other activities are being carried out. The main context of use mentioned for all the native species was their playtime in the countryside when they were children (45 %). Other contexts mentioned were: goat and sheep breeding (25 %), searching for horses (25 %), working in the field (25 %), visiting relatives or friends (20 %), searching for firewood (20 %) and/or medicinal plants (20 %). Various authors have pointed out the existence of a positive relationship between practices characterised by direct contact and everyday exploration of natural surroundings and the maintenance of traditional practices of wild plant gathering [45–47]. For example, Ladio and Lozada [30] found that in the Paineo Mapuche population those who practice livestock transhumance know and consume a higher diversity and quantity of edible wild plants compared to those who do not practice this traditional activity. Eyssartier et al. [48] found similar results in that the people who maintained horticultural practices were those who collected most medicinal wild plants. These authors have proposed that these practices are not only interconnected but that they represent possible adaptive and resilient management responses concerning the management of natural environmental resources in these rural communities of Patagonia [30, 45, 48].

The use patterns documented here support our proposal that plants with USOs are characterised by being a secondary resource which does not constitute a principal component of the diet, and is part of the “marginal use strategy” suggested for the last three centuries of rural or indigenous populations of Patagonia [20]. Ladio [19] showed that amongst inhabitants of the department of Catan-lil in Neuquen, the use of plants with USOs was very important in the past, especially as a food for children, although its current use is also sporadic. However, even though their use is secondary at the present time, these plants are even perceived as a useful emergency resource for when there is nothing else to eat. Wild emergency resources for moments of alimentary crisis are found in various societies of the world [49–51], and their permanence in the social memory is an essential aspect for the maintenance of local food security.

### Plants with USOs in the people’s cosmos

**Fig. 6:**
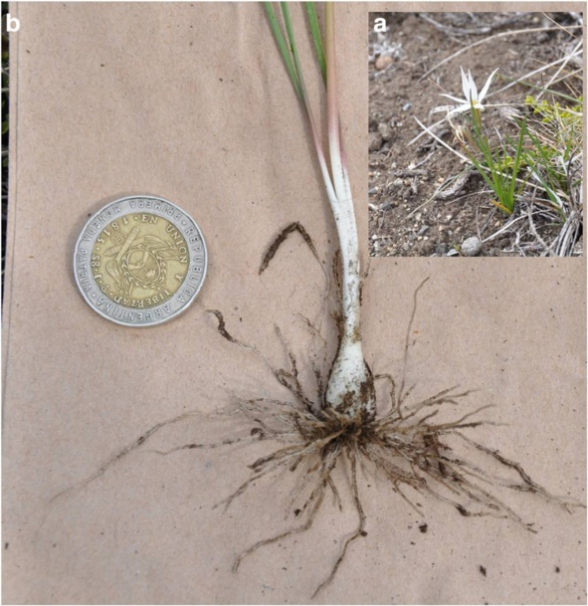
**a** General aspect of *Tristagma patagonicum*. **b** Edible bulb of *Tristagma patagonicum*

**Fig. 7:**
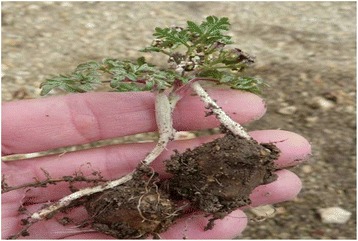
Edible tubers of *Diposis patagonica*

From the perspective of the interviewees, these plants, as with other elements of the environment they live in, are integrated into their lives due to their immediate usefulness (food or medicine), their ability to evoke childhood memories, and because they are indicators of the state of health of the environment. Of all cites, 80 % consider that consumption of these organs is of no special value from a nutritional point of view. For example, with respect to *Arjona tuberosa*: *these plants don’t build you up they only quench your thirst*…; or, *they are just sweet like candies;* referring to *Tristagma patagonica* (Fig. 6a, b))*: you have to be careful because if you eat a lot it’s bad for you; Oxalis adenophylla: it’s to trick your stomach…* A smaller proportion, however (10 % of cites), considered them to be a good food source…*because they are very healthy*…, for example, *my mother never got sick because she used to say that eating llocón was good for her…* or … *with milk it makes you strong*… This perception of good food is restricted to settlers who identify themselves as Mapuche, and make reference to *Diposis patagonica, *(Fig. 7) a species currently in use. Maybe this local perception means that at least for *Diposis patagonica* the idea of functional foods, generally defined as food having health promoting benefits in addition to their nutritional value, is prevailing for these indigenous people and is linked to their old traditions according to their long-term use as foods. A profound evaluation of this situation in combination to more nutritional and ethnopharmacological screaning are needed.

In addition to this, plants with USOs indirectly reflect the state of health of the environment in which the settlers live. For example, in various talks their presence was related to good years with a lot of rain, … *no…this year there are not many because it doesn’t rain like it used to….* The absence of these plants in their territories on other occasions is a sign of poverty, linked to the fact that… *the land is worse and worse…* due to climatic effects (short winters, prolonged droughts) or political policies, such as a reduction in their land or the inability to gain access to environments which today belong to other people: *now we don’t gather them because we don’t have them on our land any more… they are on the other side of the fence and we don’t get on well with the new owners…* It is very interesting to note that whereas for some inhabitants the practice, whether in current use or not, is considered a positive identity aspect, for example in relation to *Diposis patagonica*: *we are Mapuches, we know and respect the land… my grandmother showed me llocón and I showed it to my children…;* there was also a negative mention with respect to *Tristagma patagonicum*: *I used to eat that little onion when I was a child…my mother told me not to eat it because it was the Indians’ and it could make me sick…*

The perceptions, valuations and beliefs expressed by informants regarding the quality of USOs as a food resource, their relation with environmental aspects, access to the land and connection with identity all give meaning to the marginal use of these plants at the present time. On the other hand, their importance to Mapuche communities in terms of memories of traditional practices and of their ancestors shows the current relevance of the relational cosmovision which has characterised this people. In other works with Mapuche communities Ladio [25] found a similar relationship between cosmos and praxis, where plants with USOs, like other wild plants, have a significant role to play, are considered healthy, “good to eat” and members of the “mapu”. In this sense, it is worthy of note that one of the species cited in this work, *Diposis patagonica*, is part of a Mapuche myth, compiled by Villagrán et al. [52], which highlights its role as a food source that made subsistence possible and conferred particular characteristics of strength on the Mapuches. Nevertheless, neither this plant nor the other species were mentioned by the informants as belonging to myths, legends or stories, despite the importance of these orally-transmitted stories to the Mapuche people [53, 54].

### Cultural importance at a regional level

The species with highest cultural importance according to IC at a regional level was *Oxalis adenophylla* (Table 3). This species, which has the highest cite frequency, is known in three of the four settlements studied and is used for its medicinal, edible and ornamental properties. These variables (cite frequency, multiplicity of use and “cultivation”) are the principal characteristics influencing IC values (Table 3). *Arjona tuberosa* and *Diposis patagonica* follow; their representation in various settlements and their relative cite frequency are the variables which confer high cultural value. *Tristagma* and *Pastinaca,* still represented in three and two settlements respectively, show a very low relative cite frequency and are not used at the present time. The four remaining species were mentioned in only one settlement and cited by a low percentage of interviewees (Table 3).

These results show that at a regional level some species are more highly valued than others. The three species with highest Ic (*Oxalis*, *Arjona* and *Diposis*) are those which are currently used, while the remaining species are either not used at this time (*Tristagma*, *Pastinaca*, *Helianthus*, *Juncus*) or are used by only one person (*Azorella*).

Nevertheless, the changes which have taken place in relation to the people and species have been differential. For example, the continuous use of *Arjona tuberosa,* a native species spread widely throughout Patagonia, has been recorded from the 16th century up to the present time [20], and is known and used in all four settlements we worked in. In contrast, other native species currently used, such as *Oxalis adenophylla*, *Diposis patagonica* and *Tropaeolum porifolium* show a discontinuous historic record of use, which could be mainly due to its more restricted geographic distribution, resulting in a more local use distribution. The fact that *Pastinaca sativa* and *Helianthus tuberosus* are little known and not in current use could be due to the fact that they have only recently been incorporated in some areas of Patagonia, and therefore have limited distribution and have had less time of interaction with the rural populations of Patagonia compared with native flora.

### The use of plants with USOs in relation to age, gender, family group structure and ethnic self-identification

The richness of plants with USOs mentioned per person increases significantly with informant age (*r* = 0.55 Spearman correlation *p* < 0.05). As proposed by various authors, the higher richness of species known by older inhabitants may be because experiences of learning and use are increased with the passing of time [29, 30]. Furthermore, the practice of gathering edible wild plants is associated with other traditional subsistence activities which are actively carried out in adulthood, such as livestock breeding and transhumance [25], searching for firewood and medicinal plants [45], and horticultural practices [55]; these activities all involve profound contact with and learning about the natural surroundings. Throughout the 20th and 21st centuries (during our interviewees’ lifetimes), these traditional activities have undergone changes, tending towards their abandonment and/or replacement with activities which are increasingly linked to other lifestyles and ways of thinking, characterised by less contact with and dependence on natural surroundings (scholarisation, employment in state or private institutions, economic activities associated with tourism and dependence on food and medicinal products on the market). The older interviewees are therefore those who have mostly experienced these traditional activities, whereas the younger adults have lived through more of the changes mentioned. This is reflected in the specific knowledge of certain species. For example, some species appear to be patrimony principally of the older interviewees, (i.e. *Azorella*, *Diposis*, *Juncus, Tristagma, Helianthus, Pastinaca*), while others seem to be maintained in all age groups (i.e. *Oxalis*, *Arjona*, *Tropaeolum*).

Women and men showed no differences in the average richness of species they use (Test de Mann Whitney, p: 0.45). These results coincide with those found by other authors [56–58], indicating that certain use categories, such as edible wild resources, are shared and appropriated by both genders. In a study carried out approximately 10 years ago, Lozada et al. [59] found that men and women in Cuyín Manzano have similar knowledge of medicinal and edible plants, both in number and composition. It would thus appear that this tendency is still in effect and is representative of a pattern of knowledge and use of edible wild plants which reflects equality in knowledge of this subject on the part of men and women. Taking into account that learning and the mechanisms of cultural transmission with respect to these plants occurred mainly during infancy [60], we can postulate that these learning contexts were experienced by both sexes in a similar way. On the other hand, the fact that there is no difference in gender between the people who currently gather plants with USOs may be due on the one hand to the secondary position they occupy in the alimentation of these settlers, who use them in specific contexts which are experienced equally by both sexes (for example firewood collection, medicinal plants, search for animals, or in the past, in moments of childhood play).

**Fig. 8:**
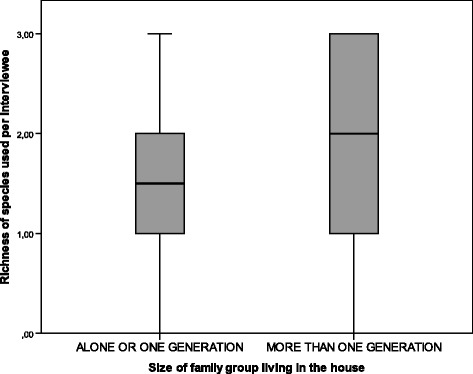
Current richness of plants with USOs used according to the family group living in the interviewee’s home (*N* = 51)

In relation to family structure, interviewees who share their homes with other generations use, on average, more plant species with USOs than those who live alone or with a partner (Mann Whitney test, *p* < 0.05; Bonferroni Test *p* < 0.0125) (Fig. 8). This supports the idea of the importance of the social environment with respect to the maintenance of use practices involving these species, in that specific contexts and needs favour their use, generally associated with health care, alimentation, play, and other types of social interaction that promote the exploration of useful resources in the environment [61, 62].

**Fig. 9:**
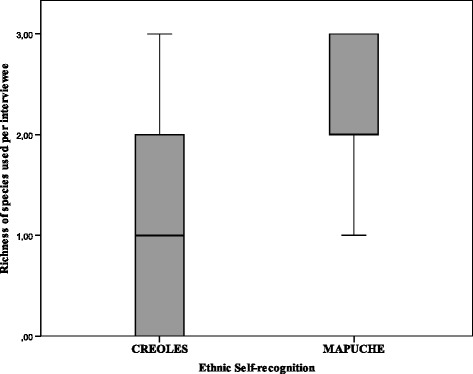
Current richness of plants with USOs used: differences between Mapuche and Creole inhabitants of the settlements studied (*N* = 51)

Finally, our results indicate that interviewees who identify themselves as belonging to the Mapuche people also use a higher average of plant species with USOs than those who do not (Mann Whitney, *p* < 0.05; Bonferroni Test *p* < 0.0125) (Fig. 9). To these native peoples wild plants have constituted both material and symbolic resources of great importance in their historical subsistence [34]. In addition, they are currently being reconfigured as elements that provide a connection with the practices of their ancestors, produce a strong link with the “earth” and become identification marks of their people’s “natural” (historical) way of life; key factors in the current political processes of identity revaluation [63].

## Conclusions

The knowledge and use of plants with USOs, studied in this work from the perspective of the corpus-cosmos-praxis complex, reveal that different resources in Patagonian communities reflect current traditions, but also the process of change. The use practices recorded here support the idea that plants with USOs lie within a pattern of marginal use, as has been true for the last centuries in Patagonia [20], following the profound socioecological transformation which has characterised human environments in this region [24, 25]. The abandonment of traditional practices which are directly linked to the use of these species, such as livestock breeding, are likely to have a negative effect on the use of plants with USOs in the communities studied. Despite their marginal use, these species generally occupy an important place in the memory (inhabitants remember their childhood and the practices of their parents and grandparents) and their presence is perceived as an indicator of environmental events (e.g., rains and drought). Unfortunately, in this kind of study it is not possible to analyse the ‘before and ‘after’ so as to establish the true trajectory of the process of loss. Nevertheless, the literature and some field data reveal that multiplicity of use is less now than it was in the past, i.e., not only are there fewer species used, but there are also fewer uses per species [30, 45]. Following Winter and Mcclatchey [64], these findings may be interpreted as a process of loss of resilience, such that the inhabitants are experiencing co-extinction of wisdom and cultural practices which could contribute diversity and flexibility to their quality of life.

In accordance, based in the local perception about the nutritional quality of *Diposis patagonica* we propose that at least this species has a prevailing role as a functional food. Future research about their plant chemical properties is necessary to evaluate this assumption. This kind of study contributes to the identification of functional aspects of wild plant use and consequently, to stimulate cultural revival and health promotion programs in the communities with their own local, cultural food.
